# Targeting Prostate Cancer Cells by an Isopeptidase Inhibitor NSC632839

**DOI:** 10.5152/tud.2025.24115

**Published:** 2025-05-21

**Authors:** Ummuhan Demir, Rabia Erdogdu

**Affiliations:** 1 Department of Molecular Biology and Genetics, Istanbul Medeniyet University Faculty of Engineering and Natural Sciences, Istanbul, Türkiye; 2 Science and Advanced Technologies Research Center (BILTAM), Istanbul Medeniyet University, Istanbul, Türkiye; 3 Department of Molecular Biology and Genetics, Biruni University Faculty of Engineering and Natural Sciences, Istanbul, Türkiye; 4 Department of Molecular Oncology, Institute of Graduate Programs, Istanbul Medeniyet University, Istanbul, Türkiye

**Keywords:** Prostate cancer, NSC632839, SENP2

## Abstract

**Objective:**

Posttranslational protein modifications are crucial for fine-tuning protein function. NSC632839 is a dual deubiquitination and desumoylation inhibitor. The desumoylation enzyme SENP2 is one of the targets of NSC632839. This study aimed to evaluate NSC632839 as an antiproliferative agent in prostate cancer (PCa).

**Methods:**

The IC50 values for NSC632839 were determined in PCa cell lines PC3 and LNCaP and normal fibroblast cells CCD-1072Sk by crystal violet staining. The colony-formation ability of PC3 and LNCaP cells upon NSC632839 treatment was evaluated by a 2D colony-formation assay. The expression level of SENP2 and its correlation with androgen receptor (AR) were investigated in PCa tissue samples using publicly available datasets.

**Results:**

The IC50 values of NSC632839 were 3.1, 1.9, and 17.7 for LNCaP, PC3, and CCD-1072Sk, respectively. In this IC50 concentration, NSC632839 completely abolished the colony-formation ability of PC3 cells. The expression level of SENP2 was elevated in metastatic PCa tissue samples and was correlated with the AR.

**Conclusion:**

NSC632839 was an antiproliferative agent in PCa cells at low doses. Therefore, NSC632839 is a strong drug candidate requiring further studies.

**Main Points**

- NSC632839, as a dual deubiquitination and desumoylation inhibitor, exhibited a significant antiproliferative effect on PCa cells (PC3 and LNCaP) in contrast to its minimal effect on normal fibroblasts.
- SENP2 is a known target of the NSC632839 small molecule inhibitor.
- SENP2 expression is significantly elevated in the late stages of PCa compared to earlier stages. SENP2 may be a potential prognostic biomarker for PCa progression.

## Introduction

The posttranslational modifications are crucial for the regulation of protein function and cellular processes. While polyubiquitination is a mark to lead protein degradation, other types of ubiquitination play roles in cellular signaling or compartmentalization. The ubiquitination process requires a series of enzymatic actions. E1 transfers the ubiquitin moiety to the E2 conjugating enzyme, and E3 ligase catalyzes the addition of ubiquitin to the target protein. K48 ubiquitination increases affinity of this target protein to the proteasome.^1^

Sumoylation is another type of posttranslational modification. Similar to ubiquitination, it requires serial action of E1, E2, and E3 enzyme activities. Posttranslational modifications are reversible processes to ensure proper regulation of protein function. Therefore, both deubiquitination and desumoylation are in order.^2^

SENP enzymes are responsible both for preprocessing and deconjugation of SUMO proteins. Seven different SENP proteins are known, each with different roles and subcellular localizations. SENP1 and SENP2 have been commonly studied in terms of their function in cancer progression among other SENP enzymes.^3^

SENP2 has a role in regulation of both fatty acid metabolism and mitochondrial function.^4^^,^^5^ P53 is also an important target of SENP2.^6^ These roles of SENP2 give a hint about the involvement of SENP2 in cancer progression.

NSC632839 is a non-selective isopeptidase inhibitor. SENP2 was identified as one of its targets.^7^ SENP proteins are designated targets for therapies in various types of cancer, especially in prostate cancer (PCa). However, the studies exploring the anti-cancer potential of SENP2 inhibitors such as NSC632839 is very limited in the literature.

In our study, the anti-cancer effect of NSC632839 in PCa was studied for the first time in the literature and also the expression level of SENP2 and its correlation with the androgen receptor (AR) in PCa tissue samples were evaluated using public databases.

## Material and Methods

### Small Molecule Inhibitor

NSC632839 was obtained from MedChemExpress (cat. no. HY-101491) and dissolved in dimethyl sulfoxide (DMSO) to obtain a 20 mM stock solution.

### Cell Culture

Androgen receptor negative PCa cells PC3 and AR positive PCa cells LNCaP were used. Normal fibroblastic CCD-1072Sk cells served as normal control cells. PC3 and LNCaP cell lines were cultured in RPMI medium (Gibco, Grand Island, NY, USA) containing 10% FBS (Gibco) and 1% penicillin/streptomycin antibiotics (Gibco), while CCD-1072Sk cells were grown in DMEM medium (Gibco, Grand Island, NY, USA) containing 10% FBS and 1% penicillin/streptomycin antibiotics.

### Cell Viability Analysis

PC3, LNCaP, and CCD-1072Sk cell lines were seeded in triplicate onto a 96-well plate at a density of 6.0 × 10^3^, 12.0 × 10^3^, and 12.0 × 10^3^ cells/well, respectively. After 24 hours, NSC632839 was applied to the cells in increasing concentrations. The duration of the treatment was 48 hours. Crystal violet staining was performed to determine viability. Briefly, the cells were washed with 1x PBS and incubated with 0.5% crystal violet for 20 minutes at room temperature. After removal of the stain, the cells were washed serially with PBS and distilled water. Methanol was added to release the crystal violet from the cells, and the absorbance was measured at 590 nm in a plate reader. The viability values were calculated with respect to the control, and IC50 values were plotted in GraphPad.

### Colony Formation Assay

PC3 cells were seeded in 6-well plates at a density of 2 x 10^5^ cells per well. After 24 hours, the cells were treated with NSC632839 at IC50 concentration. To obtain a low density of cells, the NSC-treated cells were reseeded into 6-well plates at a density of 300 cells per well after 24 hours. The cells were allowed to form colonies for 10 days by refreshing the medium in between. When the colonies became visible, they were stained with 0.5% crystal violet. Images were taken, and the number of colonies was quantified using ImageJ.

### Analysis of SENP2 Expression

The data for PCa were downloaded from GEO and cBioportal^8^ databases and analyzed with the GraphPad program.

### Statistical Analysis

All in vitro experiments were repeated at least 3 times. The significance was evaluated with a Student *t*-test between 2 groups and one-way ANOVA for more than 2 groups when appropriate. *P* < .05 was accepted as significant.

### Ethics Statement

The author declares that no human or animal subjects were used in this study. Therefore, informed consent or ethics committee approval is not required.

## Results

**Figure 1.**
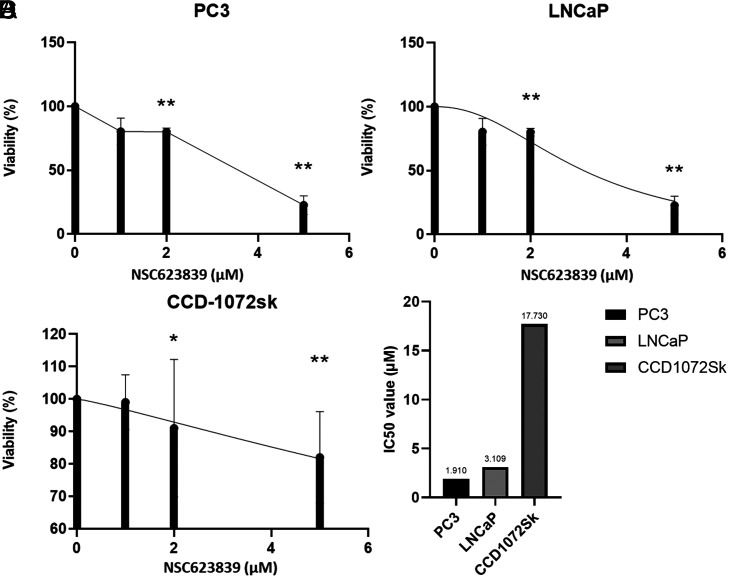
The effect of NSC632839 on PCa proliferation in vitro. (A) Dose-dependent effect of NSC632839 on PC3 cells. (B) Dose-dependent effect of NSC632839 on LNCaP cells. (C) Dose-dependent effect of NSC632839 on CCD-1072Sk cells. (D) IC50 values of NSC632839 for each cell line. Statistically significant differences are given as a result of Student’s *t*-test: ^*^P < .05, ^**^P < .01, ^***^P < .001.

To examine the effects of NSC632839 on PCa cell proliferation in vitro, human PC3 and LNCaP PCa cell lines and CCD-1072Sk cells as a control group were treated with NSC632839. After the 48-hour incubation period, cell proliferation was assessed. The effects of NSC632839 on PC3, LNCaP, and CCD-1072Sk cell proliferation were analyzed. As shown in Figure 1, treatment with NSC632839 significantly suppressed the proliferation of both PC3 and LNCaP PCa cells in a dose-dependent manner. The IC50 values were calculated as 1.9 µM, 3.1 µM and 17.7 µM for PC3 and LNCaP PCa, and CCD-1072Sk cell lines, respectively, to quantify the antiproliferative potency of NSC632839.

**Figure 2.**
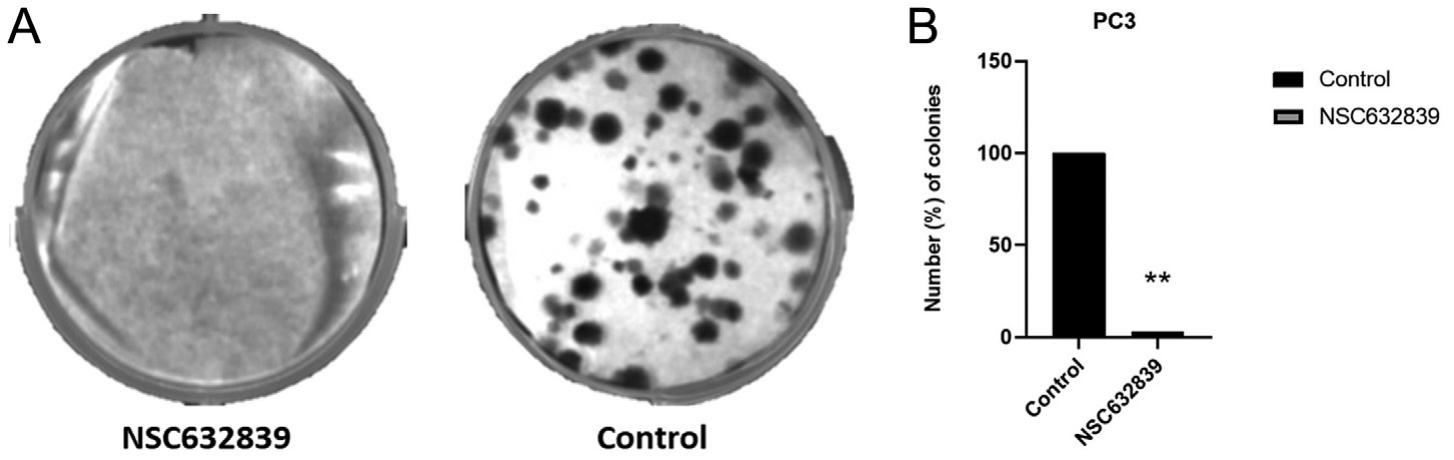
The effect of NSC632839 on clonogenic potential. (A) Control and NSC632839-treated PC3 colonies. (B) Number (%) of NSC632839 treated PC3 colonies. Statistically significant differences are given as a result of Student’s *t*-test: ^*^P < .05, ^**^P < .01, ^***^P < .001.

To evaluate the effect of NSC632839 on the colony-forming ability of PCa cells, a 2D colony formation assay was performed. PC3 cells were seeded and treated with the IC50 concentration of NSC632839. The control group received DMSO treatment. After the treatment period, the 2D colonies formed by the PC3 cells (Figure 2) were counted using ImageJ software. According to the results, there were no colonies observed in the treated groups in contrast to the control group treated with DMSO. The complete absence of colonies in the treated group proves that NSC632839 is able to completely abolish the ability of PC3 cells to form 2D colonies. This finding indicates that the NSC632839 compound has a potent inhibitory effect on the clonogenic potential of the PC3 cells.

**Figure 3.**
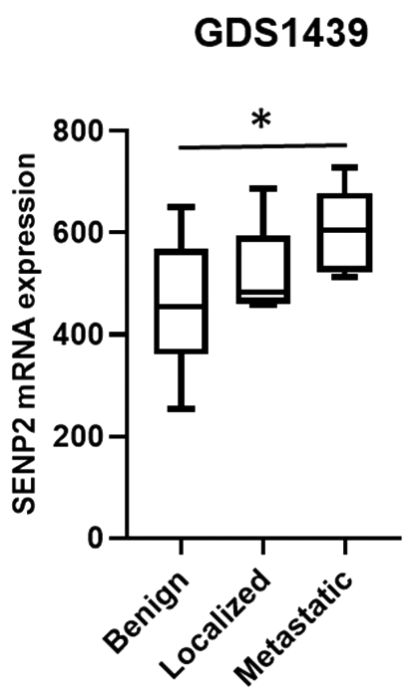
mRNA expression profile of SENP2 according to PCa stages. SENP2 expression level is given for GDS1439 data set that contains 3 class of patients, including benign, localized, and metastatic.

**Figure 4.**
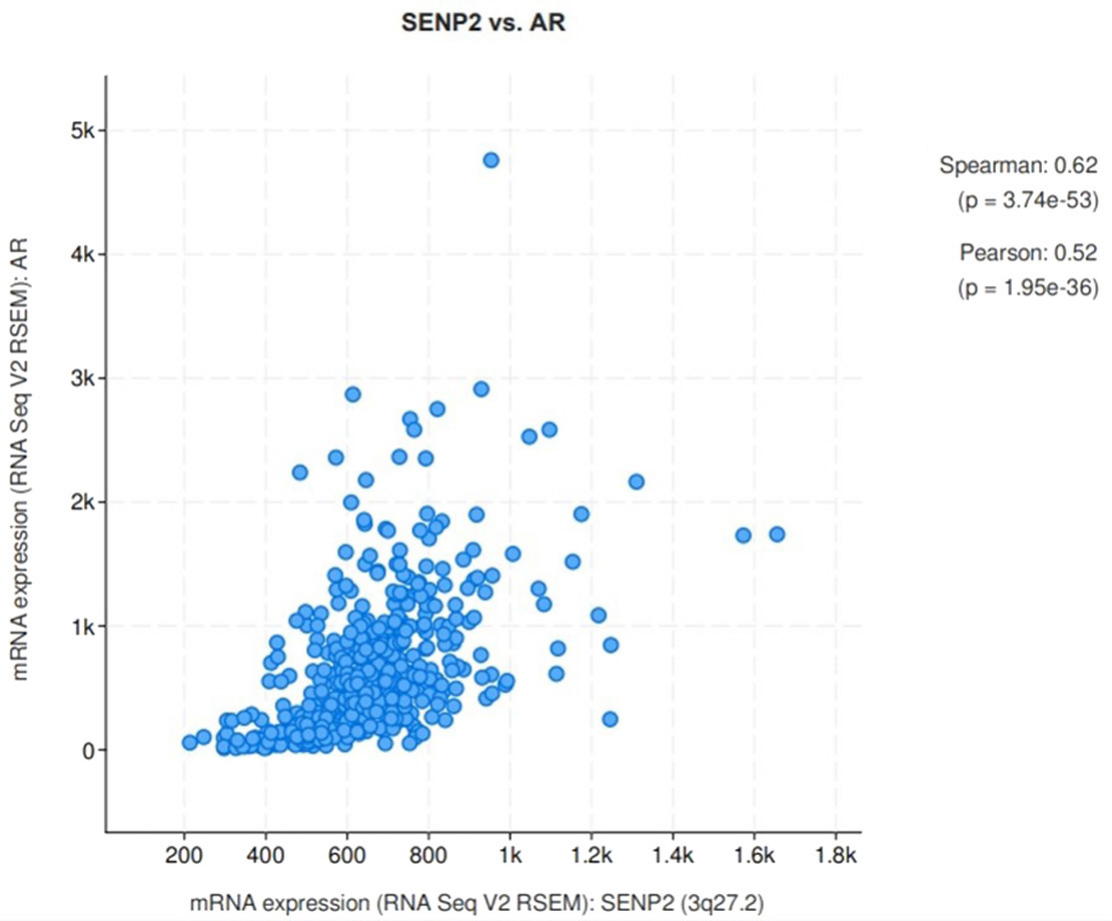
Coexpression of AR and SENP2. A significant correlation between AR expression and SENP2 expression was obtained from the cBioPortal database.

To further investigate the relationship between SENP2 mRNA expression and PCa progression, we queried the NCBI Gene Expression Omnibus (GEO) database (https://www.ncbi.nlm.nih.gov/geo/) and identified a dataset (GDS1439) that contains mRNA expression measurements from PCa patients. By analyzing the GDS1439 data, it was revealed that SENP2 expression was significantly increased as the disease progresses from benign to metastatic stage (Figure 3). Lastly, a significant positive correlation between AR and SENP2 expression was obtained from an analysis of the cBioPortal TCGA database (http://www.cbioportal.org) (Figure 4). As AR is a crucial protein in PCa, these findings suggest that SENP2 could be a potential biomarker related to the progression of PCa.

## Discussion

In our study, the antiproliferative effect of NSC was shown in PCa cells for the first time. NSC632839 diminished cell proliferation selectively in cancer cell lines compared to normal cells. This study provides evidence that the NSC632839 small molecule exerts antiproliferative and anti-clonogenic effects on PCa cells, potentially through modulation of the SENP2 pathway. The estimated correlation between SENP2 and AR expression further supports the view that SENP2 could be a promising target for PCa therapeutics and disease monitoring.

The studies about NSC632839 is very limited, and these studies have been evaluating the apoptotic,^9^ autophagic^10^ potential of this inhibitor and also its potential as a deubiquitinating enzyme inhibitor.^11^^-^ ^14^ Only study about NSC632839 on PCa cells PC3 and Du-145 was by Gupta-Saraf et al. In that study, the effect of the oncolytic virus mammalian orthoreovirus (MRV) on HIF1α stability was evaluated. It was reported that when the cells were treated with NSC632839 as a deubiquitination inhibitor, HIF1α stayed stable, proving that MRV led to HIF1α degradation via the proteasome system.^12^ Therefore, our study, not only in PCa but also for all cancer types, is the first study directly analyzing the anti-cancer potential of NSC632839 in the literature.

It was reported that NSC632839 is a dual deubiquitination and desumoylation inhibitor. SENP2 was recognized as a target at low concentration.^7^ The concentration we used in our study was quite low. Therefore, SENP2 was considered the relavant target of NSC in PCa cells, rather than the deubiquitinases USP2 and USP7.

NSC632839 was identified as an inducer of apoptosis in the absence of functional Caspase 9 by Aleo et al. They reported that NSC632839 exerted apoptotic activity by interfering with the ubiquitin-proteasome system, acting as an isopeptidase inhibitor.^9^ In 2008, Nicholson et al.^7^ determined that NSC632839 was not only a deubiquitinase but also a desumoylase inhibitor. Its activity against USP2, USP7, and SENP2 was at 45, 37, and 9.8 µM, respectively. In our study, the IC50 values obtained for cancer cells PC3 and LNCaP were 1.9 and 3.1 µM, respectively.

It was already revealed that SENP1 and SENP2 deconjugate SUMO protein from AR. However, AR transcriptional activity was regulated more efficiently by SENP1.^15^ In our study, a strong correlation was shown between the expression levels of AR and SENP2. Therefore, SENP2 may play a role in the stability of AR by desumoylation.

In conclusion, the present study combined in vitro experiments and bioinformatics analyses to elucidate the antiproliferative and anti-clonogenic potential of the NSC632839 small molecule inhibitor and identified the expression profile of SENP2 as a relevant target in PCa disease progression. Findings from in vitro studies indicate that NSC632839 exhibited a significant antiproliferative effect on PCa cell lines, in contrast to its minimal effect on normal fibroblast cells. Further analysis revealed that the NSC632839 small molecule potently suppressed the colony-forming capacity of PC3 cells, suggesting it can be a candidate drug against PCa. The study then examined the expression levels of the SENP2 gene across the PCa disease spectrum by querying gene expression databases such as the GEO. The results suggested that SENP2 exhibits significantly elevated expression in the late stages of PCa relative to the earlier disease stages. This suggests that SENP2 may hold promise as a potential prognostic biomarker for PCa progression. However, the limitation of this study was the utilization of only one dataset for PCa tissue samples and AR correlation studies. Further functional analyses, such as migration, invasion, and apoptosis assays, will be warranted to analyze the effect of NSC632839 on PCa cells.

## Data Availability

The data that support the findings of this study are available upon request from the corresponding author.

## References

[1] Weathington NM Mallampalli RK. Emerging therapies targeting the ubiquitin proteasome system in cancer. J Clin Invest. 2014;124(1):6 12. (doi: 10.1172/JCI71602) 24382383 PMC3871250

[2] Kumar A Zhang KY. Advances in the development of SUMO specific protease (SENP) inhibitors. Comp Struct Biotechnol J. 2015;13:204 211. (doi: 10.1016/j.csbj.2015.03.001) PMC4397505 25893082

[3] Tokarz P Woźniak K. SENP proteases as potential targets for cancer therapy. Cancers. 2021;13(9):2059. (doi: 10.3390/cancers13092059) PMC8123143 33923236

[4] Koo YD Choi JW Kim M SUMO-specific protease 2 (SENP2) is an important regulator of fatty acid metabolism in skeletal muscle. Diabetes. 2015;64(7):2420 2431. (doi: 10.2337/db15-0115) 25784542 PMC4477359

[5] Liu Y Liu K Thorne RF Mitochondrial SENP2 regulates the assembly of SDH complex under metabolic stress. Cell Rep. 2023;42(2):112041. (doi: 10.1016/j.celrep.2023.112041) 36708515

[6] Jiang M Chiu SY Hsu W. SUMO-specific protease 2 in Mdm2-mediated regulation of p53. Cell Death Differ. 2011;18(6):1005 1015. (doi: 10.1038/cdd.2010.168) 21183956 PMC3081924

[7] Nicholson B Leach CA Goldenberg SJ Characterization of ubiquitin and ubiquitin‐like‐protein isopeptidase activities. Protein Sci. 2008;17(6):1035 1043. (doi: 10.1110/ps.083450408) 18424514 PMC2386736

[8] Cerami E Gao J Dogrusoz U The cBio cancer genomics portal: an open platform for exploring multidimensional cancer genomics data. Cancer Discov. 2012;2(5):401 404. (doi: 10.1158/2159-8290.CD-12-0095) 22588877 PMC3956037

[9] Aleo E Henderson CJ Fontanini A Solazzo B Brancolini C. Identification of new compounds that trigger apoptosome-independent caspase activation and apoptosis. Cancer Res. 2006;66(18):9235 9244. (doi: 10.1158/0008-5472.CAN-06-0702) 16982768

[10] Yan C Huo H Yang C Zhang T Chu Y Liu Y. Ubiquitin C-terminal hydrolase L1 regulates autophagy by inhibiting autophagosome formation through its deubiquitinating enzyme activity. Biochem Biophys Res Commun. 2018;497(2):726 733. (doi: 10.1016/j.bbrc.2018.02.140) 29462615

[11] Nguyen TV. USP15 antagonizes CRL4CRBN-mediated ubiquitylation of glutamine synthetase and neosubstrates. Proc Natl Acad Sci U S A. 2021;118(40):e2111391118. (doi: 10.1073/pnas.2111391118) PMC8501880 34583995

[12] Gupta-Saraf P Miller CL. HIF-1α downregulation and apoptosis in hypoxic prostate tumor cells infected with oncolytic mammalian orthoreovirus. Oncotarget. 2014;5(2):561 574. (doi: 10.18632/oncotarget.1767) 24504474 PMC3964229

[13] Padmanabhan A Candelaria N Wong KK USP15-dependent lysosomal pathway controls p53-R175H turnover in ovarian cancer cells. Nat Commun. 2018;9(1):1270. (doi: 10.1038/s41467-018-03599-w) PMC5871815 29593334

[14] Galant C Marchandise J Stoenoiu MS Overexpression of ubiquitin-specific peptidase 15 in systemic sclerosis fibroblasts increases response to transforming growth factor β. Rheumatology (Oxford). 2019;58(4):708 718. (doi: 10.1093/rheumatology/key401) 30608617 PMC6434377

[15] Kaikkonen S Jääskeläinen T Karvonen U SUMO-specific protease 1 (SENP1) reverses the hormone-augmented SUMOylation of androgen receptor and modulates gene responses in prostate cancer cells. Mol Endocrinol. 2009;23(3):292 307. (doi: 10.1210/me.2008-0219) 19116244 PMC5428156

